# Social Networking Sites and Addiction: Ten Lessons Learned

**DOI:** 10.3390/ijerph14030311

**Published:** 2017-03-17

**Authors:** Daria J. Kuss, Mark D. Griffiths

**Affiliations:** Psychology Department, International Gaming Research Unit, Nottingham Trent University, 50 Shakespeare Street, Nottingham NG1 4FQ, UK; mark.griffiths@ntu.ac.uk

**Keywords:** social networking sites, addiction, social media, FOMO, nomophobia, smartphone addiction, microblogging, gaming, dating, recommendations

## Abstract

Online social networking sites (SNSs) have gained increasing popularity in the last decade, with individuals engaging in SNSs to connect with others who share similar interests. The perceived need to be online may result in compulsive use of SNSs, which in extreme cases may result in symptoms and consequences traditionally associated with substance-related addictions. In order to present new insights into online social networking and addiction, in this paper, 10 lessons learned concerning online social networking sites and addiction based on the insights derived from recent empirical research will be presented. These are: (i) social networking and social media use are not the same; (ii) social networking is eclectic; (iii) social networking is a way of being; (iv) individuals can become addicted to using social networking sites; (v) *Facebook* addiction is only one example of SNS addiction; (vi) fear of missing out (FOMO) may be part of SNS addiction; (vii) smartphone addiction may be part of SNS addiction; (viii) nomophobia may be part of SNS addiction; (ix) there are sociodemographic differences in SNS addiction; and (x) there are methodological problems with research to date. These are discussed in turn. Recommendations for research and clinical applications are provided.

## 1. Introduction

The history of social networking sites (SNSs) dates back to 1997, when the first SNS *SixDegrees* emerged as a result of the idea that individuals are linked via six degrees of separation [1], and is conceived as “the small world problem” in which society is viewed as becoming increasingly inter-connected [2]. In 2004, *Facebook*, was launched as an online community for students at Harvard University and has since become the world’s most popular SNS [3]. In 2016, there were 2.34 billion social network users worldwide [4]. In the same year, 22.9% of the world population used *Facebook* [5]. In 2015, the average social media user spent 1.7 h per day on social media in the USA and 1.5 h in the UK, with social media users in the Philippines having the highest daily use at 3.7 h [6]. This suggests social media use has become an important leisure activity for many, allowing individuals to connect with one another online irrespective of time and space limitations.

It is this kind of connecting or the self-perceived constant need to connect that has been viewed critically by media scholars. Following decades of researching technology-mediated and online behaviors, Turkle [7] claims overreliance on technology has led to an impoverishment of social skills, leaving individuals unable to engage in meaningful conversations because such skills are being sacrificed for constant connection, resulting in short-term attention and a decreased ability to retain information. Individuals have come to be described as “alone together”: always connected via technology, but in fact isolated [8]. The perceived need to be online may lead to compulsive use of SNSs, which in extreme cases may result in symptoms and consequences traditionally associated with substance-related addictions. Since the publication of the first ever literature review of the empirical studies concerning SNS addiction in 2011 [3], the research field has moved forward at an increasingly rapid pace. This hints at the scientific community’s increasing interest in problematic and potentially addictive social networking use. In order to present new insights into online social networking and addiction, in this paper, 10 lessons learned concerning online social networking sites and addiction based on the insights derived from recent empirical research will be presented. These are: (i) social networking and social media use are not the same; (ii) social networking is eclectic; (iii) social networking is a way of being; (iv) individuals can become addicted to using social networking sites; (v) *Facebook* addiction is only one example of SNS addiction; (vi) fear of missing out (FOMO) may be part of SNS addiction; (vii) smartphone addiction may be part of SNS addiction; (viii) nomophobia may be part of SNS addiction; (ix) there are sociodemographic differences in SNS addiction; and (x) there are methodological problems with research to date. These are discussed in turn.

## 2. 10 Lessons Learned from Recent Empirical Literature

### 2.1. Social Networking and Social Media Use Are Not the Same

Social networking and social media use have often been used interchangeably in the scientific literature. However, they are not the same. Social media refers to the web 2.0 capabilities of producing, sharing, and collaborating on content online (i.e., user-generated content, implying a social element). Accordingly, social media use includes a wide range of social applications, such as collaborative projects, weblogs, content communities, social networking sites, virtual game worlds, and virtual social worlds [9], each of which will be addressed below.

Collaborative projects can be shared and worked on jointly and simultaneously using cloud-based computing. Two different types can be distinguished: *Wikis* allow for creating, removing and modifying online content (e.g., *Wikipedia*). Social bookmarking applications, on the other hand, allow for numbers of people to accumulate and appraise websites (e.g., *Delicious*). Taken together, collaborative projects may produce a superior end result in comparison to individual projects [9], which can be linked to the concept of collective intelligence, whereby the intelligence in the group is greater than the sum of its parts [10].

Weblogs (or “blogs”) can also be considered social media. Blogs allow individuals to share personal online diaries and information (sometimes in the form of images and videos), which may or may not be commented upon by other internet users. Next, there are content communities and video-sharing sites (e.g., *YouTube*). Content may include videos, but also text (e.g., *BookCrossing*), photographs (e.g., *Instagram*), and *PowerPoint* presentations (e.g., *Slideshare*), and in most cases, there is no a need for individuals to have personal profiles, and if they do, these tend to include limited personal information. Virtual game worlds allow users to create an online alter ego in the form of an avatar and to play with other players in large gaming universes (and the next section covers gaming in more detail). Kaplan and Haenlein [9] differentiate these from virtual social worlds from virtual game worlds, whereby the former allow individuals to create online characters which live in an alternative virtual world that is similar to their real life environments on the one hand, but defies physical laws. Arguably the best example of these virtual social worlds is *Second Life*, populated by human-like avatars, who engage in activities users engage in on an everyday basis, such as furnishing houses, going shopping, and meeting friends.

Finally, there are social networking sites, which we have previously defined as “virtual communities where users can create individual public profiles, interact with real-life friends, and meet other people based on shared interests” ([3]; p. 3529). Social networking is particularly focused on connecting people, which does not apply to a number of the other social media applications outlined above. Engaging in social networking comprises a specific type of social media use, therefore they are not synonymous. Consequently, studies that have examined social media addiction and social networking addiction may also be using the terms interchangeably, suggesting nosological imprecision.

### 2.2. Social Networking Is Eclectic

Despite social networking being one type of social media use (as outlined in the previous section), the behavior is inherently eclectic because it includes a variety of apps and services that can be engaged in. For instance, social networking can be the use of traditional social networking sites, such as *Facebook. Facebook* can be considered an ‘egocentric’ SNS (rather than the previously more common virtual communities that focused on shared interests between members) because it allows individuals to represent themselves using individual profiles and wall posts. These can contain text and audiovisual content, whilst connecting to friends who often appear as real life friends and acquaintances given the main motivation of individuals to use SNSs such as *Facebook* is to maintain their connections [3].

In 2016, the most popular social networking site was *Facebook* with 1712 million active users [5]. *Facebook* has long established its supremacy in terms of active members, with membership numbers steadily increasing by 17%–20% annually [11]. *Facebook* is a very active network. Every minute, 510,000 comments are posted; 293,000 statuses are updated; and 136,000 photos are uploaded, whilst the average user spends approximately 20 min daily on the site [11].

Over the past few years, new networks have emerged that have gradually risen in popularity, particularly amongst younger generations. *Instagram* was launched in 2010 as a picture sharing SNS, claiming to “allow you to experience moments in your friends’ lives through pictures as they happen” [12]. In 2016, *Instagram* had 500 m active users [5]. *Snapchat* was launched in 2011 [13] as an SNS that allows users to message and connect with others using a smartphone and to send texts, videos, and make calls. *Snapchat* is different from other networks in that it has an inherently ephemeral nature, whereby any messages are automatically deleted shortly after the receiver has viewed them, allowing an increased experience of perceived privacy and safety online [14]. However, teenagers are especially aware of the transitory nature of *Snapchat* messages and therefore take screenshots and keep them stored on their mobile phones or in the cloud, simply to have proof of conversations and visuals spread on this medium. The privacy advantage of the medium is thereby countered. *Snapchat* had 200 million users in 2016 [5]. In the same year, *Snapchat* was the most popular SNS among 13–24 year-old adolescents and adults in the USA, with 72% of this group using them, followed by 68% *Facebook* users, and 66% *Instagram* users [15]. The popularity of *Snapchat*—particularly among young users—suggests the SNS landscape is changing in this particular demographic, with users being more aware of potential privacy risks, enjoying the lack of social pressure on *Snapchat* as well as the increased amount of control over who is viewing their ephemeral messages. However, it could also be the case that this may lead to the complete opposite by increasing the pressure to be online all the time because individuals risk missing the connecting thread in a continuing stream of messages within an online group. This may be especially the case in *Snapchat* groups/rooms created for adolescents in school or other contexts. This can lead to decreasing concentration during preparation tasks for school at home, and may lead to constant distraction because of the pressure to follow what is going on as well as the fear of missing out. From a business point of view, *Snapchat* has been particularly successful due to its novel impermanent approach to messaging, with *Facebook* founder Mark Zuckerberg offering $3 billion to buy the SNS, which has been declined by Evan Spiegel, *Snapchat’s* CEO and co-founder [13]. These facts suggest the world of traditional SNS is changing.

Social networking can be instant messaging. The most popular messaging services to date are *WhatsApp* and *Facebook Messenger* with 1000 million active users each [5]. *WhatsApp* is a mobile messaging site that allows users to connect to one another via messages and calls using their internet connection and mobile data (rather than minutes and texts on their phones), and was bought by *Facebook* in 2014 for $22 billion [16], leading to controversies about *Facebook’s* data sharing practices (i.e., *Whatsapp* phone numbers being linked with *Facebook* profiles), resulting in the European Commission fining *Facebook* [17]. In addition to *WhatsApp*, *Facebook* owns their own messaging system, which is arguably the best example of the convergence between traditional SNS use and messaging, and which functions as an app on smartphones separate from the actual *Facebook* application.

Social networking can be microblogging. Microblogging is a form of more traditional blogging, which could be considered a personal online diary. Alternatively, microblogging can also be viewed as an amalgamation of blogging and messaging, in such a way that messages are short and intended to be shared with the writer’s audience (typically consisting of ‘followers’ rather than ‘friends’ found on *Facebook* and similar SNSs). A popular example of a microblogging site is *Twitter*, which allows 140 characters per Tweet only. In 2016, *Twitter* had 313 million active users [5], making it the most successful microblogging site to date. *Twitter* has become particularly used as political tool with examples including its important role in the Arab Spring anti-government protests [18], as well as extensive use by American President Donald Trump during and following his presidential campaign [19]. In addition to microblogging politics, research has also assessed the microblogging of health issues [20].

Social networking can be gaming. Gaming can arguably be considered an element of social networking if the gaming involves connecting with people (i.e., via playing together and communicating using game-inherent channels). It has been argued that large-scale internet-enabled games (i.e., Massively Multiplayer Role-Playing Games [MMORPGs]), such as the popular *World of Warcraft*, are inherently social games situated in enormous virtual worlds populated by thousands of gamers [21,22], providing gamers various channels of communication and interaction, and allowing for the building of relationships which may extend beyond the game worlds [23]. By their very nature, games such as MMORPGs are “particularly good at simultaneously tapping into what is typically formulated as game/not game, social/instrumental, real/virtual. And this mix is *exactly* what is evocative and hooks many people. The innovations they produce there are a result of MMOGs as vibrant sites of culture” [24]. Not only do these games offer the possibility of communication, but they provide a basis for strong bonds between individuals when they unite through shared activities and goals, and have been shown to facilitate and increase intimacy and relationship quality in couples [25] and online gamers [22,23]. In addition to inherently social MMORPGs, *Facebook*-enabled games—such as *Farmville* or *Texas Hold “Em Poker*”—can be subsumed under the social networking umbrella if they are being used in order to connect with others (rather than for solitary gaming purposes) [26,27].

Social networking can be online dating. Presently, there are many online dating websites available, which offer their members the opportunity to become part of virtual communities, and they have been especially designed to meet the members’ romantic and relationship-related needs and desires [28]. On these sites, individuals are encouraged to create individual public profiles, to interact and communicate with other members with the shared interest of finding a ‘date’ and/or long-term relationships, therewith meeting the present authors’ definition of SNS. In that way, online dating sites can be considered social networking sites. However, these profiles are often semi-public, with access granted only to other members of these networks and/or subscribers to the said online dating services. According to the US think tank Pew Research Center’s Internet Project [29], 38% of singles in the USA have made use of online dating sites or mobile dating applications. Moreover, nearly 60% of internet users think that online dating is a good way to meet people, and the percentage of individuals who have met their romantic partners online has seen a two-fold increase over the last years [29]. These data suggest online dating is becoming increasingly popular, contributing to the appeal of online social networking sites for many users across the generations. However, it can also be argued that online dating sites such as *Tinder* may be less a medium for ‘long-term relationships’, given that *Tinder* use can lead to sexual engagement. This suggests the uses and gratifications perspective underlying *Tinder* use points more in the direction of other motives, such as physical and sexual aspirations and needs, rather than purely romance.

Taken together, this section has argued that social networking activities can comprise a wide variety of usage motivations and needs, ranging from friendly connection over gaming to romantic endeavors, further strengthening SNS’ natural embeddedness in many aspects of the everyday life of users. From a social networking addiction perspective, this may be similar to the literature on Internet addiction which often delineates between addictions to specific applications on the Internet (e.g., gaming, gambling, shopping, sex) and more generalized Internet addiction (e.g., concerning problematic over-use of the Internet comprising many different applications) [30,31].

### 2.3. Social Networking Is a Way of Being

In the present day and age, individuals have come to live increasingly mediated lives. Nowadays, social networking does not necessarily refer to what we do, but who we are and how we relate to one another. Social networking can arguably be considered a way of being and relating, and this is supported by empirical research. A younger generation of scholars has grown up in a world that has been reliant on technology as integral part of their lives, making it impossible to imagine life without being connected. This has been referred to as an ‘always on’ lifestyle: “It’s no longer about on or off really. It’s about living in a world where being networked to people and information wherever and whenever you need it is just assumed” [32]. This has two important implications. First, being ‘on’ has become the status quo. Second, there appears to be an inherent understanding or requirement in today’s technology-loving culture that one needs to engage in online social networking in order not to miss out, to stay up to date, and to connect. Boyd [32] herself refers to needing to go on a “digital sabbatical” in order not be on, to take a vacation from connecting, with the caveat that this means still engaging with social media, but deciding which messages to respond to.

In addition to this, teenagers particularly appear to have subscribed to the cultural norm of continual online networking. They create virtual spaces which serve their need to belong, as there appear to be increasingly limited options of analogous physical spaces due to parents’ safety concerns [33]. Being online is viewed as safer than roaming the streets and parents often assume using technology in the home is normal and healthy, as stated by a psychotherapist treating adolescents presenting with the problem of Internet addiction: “Use of digital media is the culture of the household and kids are growing up that way more and more” [34]. Interestingly, recent research has demonstrated that sharing information on social media increases life satisfaction and loneliness for younger adult users, whereas the opposite was true for older adult users [35], suggesting that social media use and social networking are used and perceived very differently across generations. This has implications for social networking addiction because the context of excessive social networking is critical in defining someone as an addict, and habitual use by teenagers might be pathologized using current screening instruments when in fact the activity—while excessive—does not result in significant detriment to the individual’s life [36].

SNS use is also driven by a number of other motivations. From a uses and gratifications perspective, these include information seeking (i.e., searching for specific information using SNS), identity formation (i.e., as a means of presenting oneself online, often more favorably than offline) [37], and entertainment (i.e., for the purpose of experiencing fun and pleasure) [38]. In addition to this, there are the motivations such as voyeurism [39] and cyberstalking [40] that could have potentially detrimental impacts on individuals’ health and wellbeing as well as their relationships.

It has also been claimed that social networking meets basic human needs as initially described in Maslow’s hierarchy of needs [41]. According to this theory, social networking meets the needs of safety, association, estimation, and self-realization [42]. Safety needs are met by social networking being customizable with regards to privacy, allowing the users to control who to share information with. Associative needs are fulfilled through the connecting function of SNSs, allowing users to ‘friend’ and ‘follow’ like-minded individuals. The need to estimate is met by users being able to ‘gather’ friends and ‘likes’, and compare oneself to others, and is therefore related to Maslow’s need of esteem. Finally, the need for self-realization, the highest attainable goal that only a small minority of individuals are able to achieve, can be reached by presenting oneself in a way one wants to present oneself, and by supporting ‘friends’ on those SNSs who require help. Accordingly, social networking taps into very fundamental human needs by offering the possibilities of social support and self-expression [42]. This may offer an explanation for the popularity of and relatively high engagement with SNSs in today’s society. However, the downside is that high engagement and being always ‘on’ or engaged with technology has been considered problematic and potentially addictive in the past [43], but if being ‘always on’ can be considered the status quo and most individuals are ‘on’ most of the time, where does this leave problematic use or addiction? The next section considers this question.

### 2.4. Individuals Can Become Addicted to Using Social Networking Sites

There is a growing scientific evidence base to suggest excessive SNS use may lead to symptoms traditionally associated with substance-related addictions [3,44]. These symptoms have been described as salience, mood modification, tolerance, withdrawal, relapse, and conflict with regards to behavioral addictions [45], and have been validated in the context of the Internet addiction components model [46]. For a small minority of individuals, their use of social networking sites may become the single most important activity that they engage in, leading to a preoccupation with SNS use (salience). The activities on these sites are then being used in order to induce mood alterations, pleasurable feelings or a numbing effect (mood modification). Increased amounts of time and energy are required to be put into engaging with SNS activities in order to achieve the same feelings and state of mind that occurred in the initial phases of usage (tolerance). When SNS use is discontinued, addicted individuals will experience negative psychological and sometimes physiological symptoms (withdrawal), often leading to a reinstatement of the problematic behavior (relapse). Problems arise as a consequence of the engagement in the problematic behavior, leading to intrapsychic (conflicts within the individual often including a subjective loss of control) and interpersonal conflicts (i.e., problems with the immediate social environment including relationship problems and work and/or education being compromised).

Whilst referring to an ‘addiction’ terminology in this paper, it needs to be noted that there is much controversy within the research field concerning both the possible overpathologising of everyday life [47,48] as well as the most appropriate term for the phenomenon. On the one hand, current behavioral addiction research tends to be correlational and confirmatory in nature and is often based on population studies rather than clinical samples in which psychological impairments are observed [47]. Additional methodological problems are outlined below (Section 2.10). On the other hand, in the present paper, the present authors do not discriminate between the label addiction, compulsion, problematic SNS use, or other similar labels used because these terms are being used interchangeably by authors in the field. Nevertheless, when referring to ‘addiction’, the present authors refer to the presence of the above stated criteria, as these appear to hold across both substance-related as well as behavioral addictions [45] and indicate the requirement of significant impairment and distress on behalf of the individual experiencing it in order to qualify for using clinical terminology [49], such as the ‘addiction’ label.

The question then arises as what it is that individuals become addicted to. Is it the technology or is it more what the technology allows them to do? It has been argued previously [34,50] that the technology is but a medium or a tool that allows individuals to engage in particular behaviors, such as social networking and gaming, rather than being addictive *per se*. This view is supported by media scholars: “To an outsider, wanting to be always-on may seem pathological. All too often it’s labelled an addiction. The assumption is that we’re addicted to the technology. The technology doesn’t matter. It’s all about the people and information” [32]. Following this thinking, one could claim that it is not an addiction to the technology, but to connecting with people, and the good feelings that ‘likes’ and positive comments of appreciation can produce. Given that connection is the key function of social networking sites as indicated above, it appears that ‘social networking addiction’ may be considered an appropriate denomination of this potential mental health problem.

There are a numbers of models which offer explanations as to the development of SNS addiction [51]. According to the cognitive-behavioral model, excessive social networking is the consequence of maladaptive cognitions and is exacerbated through a number of external issues, resulting in addictive use. The social skill model suggests individuals use SNSs excessively as a consequence of low self-presentation skills and preference for online social interaction over face-to-face communication, resulting in addictive SNS use [51]. With respect to the socio-cognitive model, excessive social networking develops as a consequence of positive outcome expectations, Internet self-efficacy, and limited Internet self-regulation, leading to addictive SNS use [51]. It has furthermore been suggested that SNS use may become problematic when individuals use it in order to cope with everyday problems and stressors, including loneliness and depression [52]. Moreover, it has been contended that excessive SNS users find it difficult to communicate face-to-face, and social media use offers a variety of immediate rewards, such as self-efficacy and satisfaction, resulting in continued and increased use, with the consequence of exacerbating problems, including neglecting offline relationships, and problems in professional contexts. The resultant depressed moods are then dealt with by continued engagement in SNSs, leading to a vicious cycle of addiction [53]. Cross-cultural research including 10,930 adolescents from six European countries (Greece, Spain, Poland, the Netherlands, Romania, and Iceland) furthermore showed that using SNS for two or more hours a day was related to internalizing problems and decreased academic performance and activity [54]. In addition, a study using a sample of 920 secondary school students in China indicated neuroticism and extraversion predicted SNS addiction, clearly differentiating individuals who experience problems as a consequence of their excessive SNS use from those individuals who used games or the Internet in general excessively [55], further contributing to the contention that SNS addiction appears to be a behavioral problem separate from the more commonly researched gaming addiction. In a study using a relatively small representative sample of the Belgian population (n = 1000), results suggested 6.5% were using SNSs compulsively, with this group having lower scores on measures of emotional stability and agreeableness, conscientiousness, perceived control and self-esteem, and higher scores on loneliness and depressive feelings [56].

### 2.5. Facebook Addiction Is Only One Example of SNS Addiction

Over the past few years, research in the SNS addiction field has largely focused on a potential addiction to using *Facebook* specifically, rather than other SNSs (see e.g., [57,58,59,60,61,62,63,64,65]). However, recent research suggests individuals may develop addiction-related problems as a consequence of using other SNSs, such as *Instagram* [66]. It has been claimed that users may experience gratification through sharing photos on *Instagram*, similar to the gratification they experience when using *Facebook*, suggesting that the motivation to share photos can be explained by uses and gratifications theory [66,67]. This may also be the reason for why individuals have been found to be less likely to experience addiction-related symptoms when using *Twitter* in contrast to *Instagram* [66]. In addition to the gratification received through photo sharing, these websites also allow to explore new identities [68], which may be considered to contribute to gratification, as supported by previous research [69]. Research has also suggested that *Instagram* use in particular appears to be potentially addictive in young UK adults [66], offering further support for the contention that *Facebook* addiction is only one example of SNS addiction.

Other than the presence and possible addictive qualities of SNSs other than *Facebook*, it has been contended that the respective activities which take place on these websites need to be considered when studying addiction [70]. For instance, *Facebook* users can play games such as *Farmville* [36], gamble online [71], watch videos, share photos, update their profiles, and message their friends [3]. Other researchers have moved beyond the actual website use that is referred to in these types of addictions, and specifically focused on the main activities individuals engage in, referring to constructs such as ‘e-communication addiction’ [72]. It has also been claimed the term ‘*Facebook* addiction’ is already obsolete as there are different types of SNSs that can be engaged in and different activities that can take place on these SNSs [70]. Following this justified criticism, researchers who had previously studied *Facebook* addiction specifically [58] have now turned to studying SNS addiction more generally instead [73], demonstrating the changing definitional parameters of social networking in this evolving field of research.

### 2.6. Fear of Missing Out (FOMO) May Be Part of SNS Addiction

Recent research [74,75] has suggested that high engagement in social networking is partially due to what has been named the ‘fear of missing out’ (FOMO). FOMO is “a pervasive apprehension that others might be having rewarding experiences from which one is absent” [76]. Higher levels of FOMO have been associated with greater engagement with *Facebook*, lower general mood, lower wellbeing, and lower life satisfaction, mixed feelings when using social media, as well as inappropriate and dangerous SNS use (i.e., in university lectures, and or whilst driving) [76]. In addition to this, research [77] suggests that FOMO predicts problematic SNS use and is associated with social media addiction [78], as measured with a scale adapted from the Internet Addiction Test [79]. It has been debated whether FOMO is a specific construct, or simply a component of relational insecurity, as observed for example with the attachment dimension of preoccupation with relationships in research into problematic Internet use [80].

In one study using 5280 social media users from several Spanish-speaking Latin-American countries [74] it was found that FOMO predicts negative consequences of maladaptive SNS use. In addition, this study also found that the relationship between psychopathology (as operationalized by anxiety and depression symptoms and assessed via the Hospital Anxiety and Depression Scale) and negative consequences of SNS use were mediated by FOMO, emphasizing the importance of FOMO in the self-perceived consequences of high SNS engagement. Moreover, other research [75] using 506 UK *Facebook* users has found that FOMO mediates the relationship between high SNS use and decreased self-esteem. Research with psychotherapists working with clients seeking help for their Internet use-related behaviors also suggested that young clients “fear the sort of relentlessness of on-going messaging (…). But concurrently with that is an absolute terror of exclusion” [34]. Taken together, these findings suggest FOMO may be a significant predictor or possible component of potential SNS addiction, a contention that requires further consideration in future research. Further work is needed into the origins of FOMO (both theoretically and empirically), as well as research into why do some SNS users are prone to FOMO and develop signs of addictions compared to those who do not.

### 2.7. Smartphone Addiction May Be Part of SNS Addiction

Over the last decade, research assessing problematic and possibly addictive mobile phone use (including smartphones) has proliferated [81], suggesting some individuals may develop addiction-related problems as a consequence of their mobile phone use. Recent research has suggested problematic mobile phone use is a multi-faceted condition, with dependent use being one of four possible pathways, in addition to dangerous, prohibited, and financially problematic use [82]. According to the pathway model, an addictive pattern of mobile phone use is characterized by the use of specific applications, including calls, instant messaging, and the use of social networks. This suggests that rather than being an addictive medium *per se*, mobile technologies including smartphones and tablets are media that enable the engagement in potentially addictive activities, including SNS use. Put another way, it could be argued that mobile phone addicts are no more addicted to their phones than alcoholics are addicted to bottles.

Similarly, it has been argued previously that individuals do not become addicted to the Internet *per se*, but to the activities they engage in on the Internet, such as gaming [50] or SNS use [3]. With the advent and ubiquity of mobile technologies, this supposition is more pertinent than ever. Using social networking sites is a particularly popular activity on smartphones, with around 80% of social media used via mobile technologies [83]. For instance, approximately 75% of *Facebook* users access the SNS via their mobile phones [84]. Therefore, it can be suggested that smartphone addiction may be part of SNS addiction. Previous research [73] supported this supposition by specifically indicating that social networking is often engaged in via phones, which may contribute to its addictive potential. Accordingly, it is necessary to move towards nosological precision, for the benefit of both individuals seeking help in professional settings, as well as research that will aid developing effective treatment approaches for those in need.

### 2.8. Nomophobia May Be Part of SNS Addiction

Related to both FOMO and mobile phone addiction is the construct of nomophobia. Nomophobia has been defined as “no mobile phone phobia”, i.e., the fear of being without one’s mobile phone [85]. Researchers have called for nomophobia to be included in the DSM-5, and the following criteria have been outlined to contribute to this problem constellation: regular and time-consuming use, feelings of anxiety when the phone is not available, “ringxiety” (i.e., repeatedly checking one’s phone for messages, sometimes leading to phantom ring tones), constant availability, preference for mobile communication over face to face communication, and financial problems as a consequence of use [85]. Nomophobia is inherently related to a fear of not being able to engage in social connections, and a preference for online social interaction (which is the key usage motivation for SNSs [3]), and has been linked to problematic Internet use and negative consequences of technology use [86], further pointing to a strong association between nomophobia and SNS addiction symptoms.

Using mobile phones is understood as leading to alterations in everyday life habits and perceptions of reality, which can be associated with negative outcomes, such as impaired social interactions, social isolation, as well as both somatic and mental health problems, including anxiety, depression, and stress [85,87]. Accordingly, nomophobia can lead to using the mobile phone in an impulsive way [85], and may thus be a contributing factor to SNS addiction as it can facilitate and enhance the repeated use of social networking sites, forming habits that may increase the general vulnerability for the experience of addiction-related symptoms as a consequence of problematic SNS use.

### 2.9. There Are Sociodemographic Differences in SNS Addiction

Research suggests there are sociodemographic differences among those addicted to social networking. In terms of gender, psychotherapists treating technology-use related addictions suggest SNS addiction may be more common in female rather than male patients, and describe this difference based on usage motivations:

(…) girls don’t play role-playing games primarily, but use social forums excessively, in order to experience social interaction with other girls and above all to feel understood in their very individual problem constellations, very different from boys, who want to experience narcissistic gratification via games. This means the girls want direct interaction. They want to feel understood. They want to be able to express themselves. (…) we’re getting girls with clinical pictures that are so pronounced that we have to admit them into inpatient treatment. (…) we have to develop strategies to specifically target girls much better because there appears a huge gap. Epidemiologically, they are a very important group, but we’re not getting them into consultation and treatment.

[34]

This quote highlights two important findings. First, in the age group of 14–16 years, girls appear to show a higher prevalence of addictions to the Internet and SNSs, as found in a representative German sample [88], and second, teenage girls may be underrepresented in clinical samples. Moreover, another study on a representative sample demonstrated that the distribution of addiction criteria varies between genders and that extraversion is a personality trait differentiating between intensive and addictive use [89].

Cross-sectional research is less conclusive as regards the contribution of gender as a risk factor for SNS addiction. A higher prevalence of *Facebook* addiction was found in a sample of 423 females in Norway using the *Facebook* Addiction Scale [58]. Among Turkish teacher candidates, the trend was reversed, suggesting males were significantly more likely to be addicted to using *Facebook* [90] as assessed via an adapted version of Young’s Internet Addiction Test [79].

In other studies, no relationship between gender and addiction was found. For instance, using a version of Young’s Internet Addiction Test modified for SNS addiction in 277 young Chinese smartphone users, gender did not predict SNS addiction [91]. Similarly, another study assessing SNS dependence in 194 SNS users did not find a relationship between gender and SNS dependence [51]. In a study of 447 university students in Turkey, *Facebook* addiction was assessed using the *Facebook* Addiction Scale, but did not find a predictive relationship between gender and *Facebook* addiction [62].

Furthermore, the relationships between gender and SNS addiction may be further complicated by other variables. For instance, recent research by Oberst et al. [74] found that only for females, anxiety and depression symptoms significantly predicted negative consequences of SNS use. The researchers explained this difference by suggesting that anxiety and depression experience in girls may result in higher SNS usage, implicating cyclical relationships in that psychopathological symptom experience may exacerbate negative consequences due to SNS use, which may then negatively impact upon perceived anxiety and depression symptoms.

In terms of age, studies indicate that younger individuals may be more likely to develop problems as a consequence of their excessive engagement with online social networking sites [92]. Moreover, research suggests perceptions as to the extent of possible addiction appear to differ across generations. A recent study by [72] found that parents view their adolescents’ online communication as more addictive than the adolescents themselves perceive it to be. This suggests that younger generations significantly differ from older generations in how they use technology, what place it has in their lives, and how problematic they may experience their behaviors to be. It also suggests that external accounts (such as those from parents in the case of children and adolescents) may be useful for clinicians and researchers in assessing the extent of a possible problem as adolescents may not be aware of the potential negative consequences that may arise as a result of their excessive online communication use. Interestingly, research also found that mothers are more likely to view their adolescents’ behavior as potentially more addictive relative to fathers, whose perception tended to be that of online communication use being less of a problem [72]. Taken together, although there appear differences in SNS addiction with regards to sociodemographic characteristics of the samples studied, such as gender, future research is required in order to clearly indicate where these differences lie specifically, given that much of current research appears somewhat inconclusive.

### 2.10. There Are Methodological Problems with Research to Date

Given that the research field is relatively young, studies investigating social networking site addiction unsurprisingly suffer from a number of methodological problems. Currently, there are few estimations of the prevalence of social networking addiction with most studies comprising small and unrepresentative samples [3]. As far as the authors are aware, only one study (in Hungary) has used a nationally representative sample. The study by Bányai and colleagues [93] reported that 4.5% of 5961 adolescents (mean age 16 years old) were categorized as ‘at-risk’ of social networking addiction using the Bergen Social Media Addiction Scale. However, most studies investigating social networking addiction use various assessment tools, different diagnostic criteria as well as varying cut-off points, making generalizations and study cross-comparisons difficult [53].

Studies have made use of several different psychometric scales and six of these are briefly described below. The Addictive Tendencies Scale (ATS) [94] is based on addiction theory and uses three items, salience, loss of control, and withdrawal, whilst viewing SNS addiction as dimensional construct. The Bergen *Facebook* Addiction Scale (BFAS) [58] is based on Griffiths’ [45] addiction components, using a polythetic scoring method (scoring 3 out of 4 on each criterion on a minimum of four of the six criteria) and has been shown to have good psychometric properties. The Bergen Social Media Addiction Scale is similar to the BFAS in that ‘*Facebook*’ is replaced with ‘Social Media’ [95]. The E-Communication Addiction Scale [72] includes 22 questions with four subscales scored on a five-point Likert scale—addressing issues such as lack of self-control (cognitive), e-communication use in extraordinary places, worries, and control difficulty (behavioral)—and it has been found to have a high internal consistency, measuring e-communication addiction across different severity levels, ranging from very low to very high.

The *Facebook* Dependence Questionnaire (FDQ) [96] uses eight items based on the Internet Addiction Scale [97], with the endorsement of five out of eight criteria signifying addiction to using *Facebook*. The Social Networking Addiction Scale (SNWAS) [51] is a five-item scale which uses Charlton and Danforth’s engagement vs. addiction questionnaire [98,99] as a basis, viewing SNS addiction as a dimensional construct. This is by no means an exhaustive list, but those assessment tools highlighted here simply demonstrate that the current social networking addiction scales are based on different theoretical frameworks and use various cut-offs, and this precludes researchers from making cross-study comparisons, and severely limits the reliability of current SNS epidemiological addiction research.

Taken together, the use of different conceptualizations, assessment instruments, and cut-off points decreases the reliability of prevalence estimates because it hampers comparisons across studies, and it also questions the construct validity of SNS addiction. Accordingly, researchers are advised to develop appropriate criteria that are clinically sensitive to identify individuals who present with SNS addiction specifically, whilst clinicians will benefit from a reliable and valid diagnosis in terms of treatment development and delivery.

## 3. Discussion

In this paper, lessons learned from the recent empirical literature on social networking and addiction have been presented, following on from earlier work [3] when research investigating SNS addiction was in its infancy. The research presented suggests SNSs have become a way of being, with millions of people around the world regularly accessing SNSs using a variety of devices, including technologies on the go (i.e., tablets, smartphones), which appear to be particularly popular for using SNSs. The activity of social networking itself appears to be specifically eclectic and constantly changing, ranging from using traditional sites such as *Facebook* to more socially-based online gaming platforms and dating platforms, all allowing users to connect based on shared interests. Research has shown that there is a fine line between frequent non-problematic habitual use and problematic and possibly addictive use of SNSs, suggesting that users who experience symptoms and consequences traditionally associated with substance-related addictions (i.e., salience, mood modification, tolerance, withdrawal, relapse, and conflict) may be addicted to using SNSs. Research has also indicated that a fear of missing out (FOMO) may contribute to SNS addiction, because individuals who worry about being unable to connect to their networks may develop impulsive checking habits that over time may develop into an addiction. The same thing appears to hold true for mobile phone use and a fear of being without one’s mobile phone (i.e., nomophobia), which may be viewed as a medium that enables the engagement in SNSs (rather than being addictive *per se*). Given that engaging in social networking is a key activity engaged in using mobile technologies, FOMO, nomophobia, and mobile phone addiction appear to be associated with SNS addiction, with possible implications for assessment and future research.

In addition to this, the lessons learned from current research suggest there are sociodemographic differences in SNS addiction. The lack of consistent findings regarding a relationship with gender may be due to different sampling techniques and various assessment instruments used, as well as the presence of extraneous variables that may contribute to the relationships found. All of these factors highlight possible methodological problems of current SNS addiction research (e.g., lack of cross-comparisons due to differences in sampling and classification, lack of control of confounding variables), which need to be addressed in future empirical research. In addition to this, research suggests younger generations may be more at risk for developing addictive symptoms as a consequence of their SNS use, whilst perceptions of SNS addiction appear to differ across generations. Younger individuals tend to view their SNS use as less problematic than their parents might, further contributing to the contention that SNS use has become a way of being and is contextual, which must be separated from the experience of actual psychopathological symptoms. The ultimate aim of research must be not to overpathologize everyday behaviors, but to carry out better quality research as this will help facilitate treatment efforts in order to provide support for those who may need it.

Based on the 10 lessons learned from recent SNS addiction research, the following recommendations are provided. First, researchers are recommended to consider including an assessment of FOMO and/or nomophobia in SNS addiction screening instruments because both constructs appear related to SNS addiction. Second, it is recommended that social networking site use is measured across different technologies with which it can be accessed, including mobile and smartphones. It is of fundamental importance to study what kinds of activities are being engaged in online (social networking, gaming, etc.), rather than the medium through which these activities are engaged in (i.e., desktop computer, tablet, mobile/smartphone). Third, risk factors associated with problematic social networking need to be assessed longitudinally to provide a clearer indication of developmental etiology, and to allow for the design of targeted prevention approaches. Fourth, clinical samples need to be included in research in order to ensure the sensitivity and specificity of the screening instruments developed. Fifth, in terms of treatment, unlike treating substance-related addictions, the main treatment goal should be control rather than abstinence. Arguably, abstinence cannot realistically be achieved in the context of SNS addiction because the Internet and social networking have become integral elements of our lives [3,8,33]. Rather than discontinuing social networking completely, therapy should focus on establishing controlled SNS use and media awareness [53].

## 4. Conclusions

This paper has outlined ten lessons learned from recent empirical literature on online social networking and addiction. Based on the presented evidence, the way forward in the emerging research field of social networking addiction requires the establishment of consensual nosological precision, so that both researchers and clinical practitioners can work together and establish productive communication between the involved parties that enable reliable and valid assessments of SNS addiction and associated behaviors (e.g., problematic mobile phone use), and the development of targeted and specific treatment approaches to ameliorate the negative consequences of such disorders.
